# NgR1 pathway expression in cerebral ischemic Sprague-Dawley rats with cognitive impairment

**DOI:** 10.22038/ijbms.2021.53316.12011

**Published:** 2021-06

**Authors:** Ju Sun, Ruifang Sun, Chao Li, Xun Luo, Jiemei Chen, Jiena Hong, Yan Zeng, Qing Mei Wang, Hongmei Wen

**Affiliations:** 1 Department of Rehabilitation Medicine, The Third Affiliated Hospital, Sun Yat-sen University, 600 Tianhe Road, Guangzhou 510630, Guangdong Province, China; 2 Department of Rehabilitation Medicine, Guangzhou Panyu Central Hospital, No.8 Fuyu east Road, Guangzhou 511400, Guangdong Province, China; 3 Kerry Rehabilitation Medicine Research Institute, Shenzhen 518048, Guangdong Province, China; 4 Shenzhen Dapeng New District Nan’ao People’s Hospital Shenzhen 518048, Guangdong Province, China; 5 Stroke Biological Recovery Laboratory, Spaulding Rehabilitation Hospital, The Teaching Affiliate of Harvard Medical School,96 13^th^ Street, Charlestown, MA 02129, USA

**Keywords:** Axons, Brain ischemia, Cognitive dysfunction, Rats, Sprague-Dawley, Stroke

## Abstract

**Objective(s):**

This study aimed to determine the effect of ischemic occlusion duration and recovery time course on motor and cognitive function, identify optimal conditions for assessing cognitive function with minimal interference from motor deficits, and elucidate the underlying mechanism of axonal inhibitors.

**Materials and Methods:**

Sprague-Dawley (SD) rats were randomly allocated to the transient middle cerebral artery occlusion (tMCAO) 60-min (tMCAO60min), tMCAO90min, tMCAO120min, and sham groups. We conducted forelimb grip strength, two-way shuttle avoidance task, and novel object recognition task (NORT)tests at three time points (14, 21, and 28 days). Expression of Nogo receptor-1 (NgR1), the endogenous antagonist lateral olfactory tract usher substance, ras homolog family member A (Rho-A), and RhoA-activated Rho kinase (ROCK) was examined in the ipsilateral thalamus.

**Results:**

There was no difference in grip strength between sham and tMCAO_90min_ rats at 28 days. tMCAO_90min_ and tMCAO_120min _rats showed lower discrimination indices in the NORT than sham rats on day 28. Compared with that in sham rats, the active avoidance response rate was lower in tMCAO_90min_ rats on days 14, 21, and 28 and in tMCAO_120min _rats on days 14 and 21. Furthermore, 50-54% of rats in the tMCAO_90min _group developed significant cognitive impairment on day 28, and thalamic NgR1, RhoA, and ROCK expression were greater in tMCAO_90min_ rats than in sham rats.

**Conclusion:**

Employing 90-min tMCAO in SD rats and assessing cognitive function 28 days post-stroke could minimize motor dysfunction effects in cognitive function assessments. Axonal inhibitor deregulation could be involved in poststroke cognitive impairment.

## Introduction

Ischemic stroke is among the major causes of mortality and the leading cause of acquired adult disability worldwide (1). The consequences of stroke include deficits in both sensorimotor and cognitive functioning (2). The prevalence of dementia is reported to be ninefold higher in patients 3 months after an ischemic stroke than in controls (3). Poststroke dementia progression cannot be completely explained as a direct consequence of primary ischemic damage (4). One-third of stroke survivors may present with dementia (5). The annual global economic cost of dementia is estimated to be approximately 81.8 billion US dollars, which accounts for 1.1% of the world’s gross domestic product. Thus, dementia could be a major obstacle to social and economic development in the future (6).

Middle cerebral artery (MCA) occlusion using an intraluminal suture is a common ischemia model in rodents given its convenience, reproducibility, reliability, and similarity to human ischemic stroke. Sensorimotor behavior and cognitive impairments have been extensively studied using this model (7, 8). However, poststroke assessment of deficits in cognitive performance could be impeded by sensorimotor impairments. Most cognitive assessments in this model involve movement and sensory function. Therefore, sensorimotor impairment can confound the interpretation of cognitive function. Furthermore, it remains unclear whether cognitive impairment can be detected in the transient MCA occlusion (tMCAO) model (9, 10). There have been few studies on the optimal duration of ischemia-reperfusion for measuring executive impairment with minimal interference from sensorimotor deficits.

Neurite outgrowth inhibitor-A (Nogo-A) is a well-known myelin-associated axon growth-inhibitory protein that has been shown to inhibit the migration and spread of nerve cells and plays an important role in preventing axon regeneration and reconnection after stroke (11). Delekate *et al*. and our group previously (12, 13) reported that Nogo-A and its receptor Nogo receptor-1 (NgR1), which trigger the downstream RhoA/ROCK signaling pathway, induce growth cone collapse and neurite outgrowth inhibition, which are associated with tMCAO-induced motor impairment. NgR1 negatively affects plasticity and cognitive recovery after traumatic brain injury; in contrast, NgR1 inhibition enhances cognitive function recovery (14). Compared with mature adults, cognitively impaired aged rats were found to have significantly increased protein expression of RhoA (15). As an endogenous NgR1 antagonist, lateral olfactory tract usher substance (LOTUS), which prevents Nogo from binding to NgR1, could be involved in cognitive function (16-18). NgR1-antagonizing protein expression decreases with aging and cognitive decline (19). It remains unclear whether the NgR1/RhoA/ROCK pathway and endogenous antagonists are involved in cognitive function impairment after cerebral ischemia.

In this study, we hypothesized that the thalamic NgR1/RhoA/ROCK pathway is associated with cognitive dysfunction in tMCAO rats. We aimed to determine the optimal protocol for assessing cognitive impairment, as measured by the novel object recognition task (NORT) and two-way shuttle box test, with minimal interference from sensorimotor deficits in SD rats, to provide evidence for selecting MCA occlusion duration and behavioral test time points, especially cognitive function. Furthermore, we aimed to investigate NgR1 pathway expression in the optimal rodent model of cognitive impairment.

## Materials and Methods

***Animals***

Adult male Sprague-Dawley (SD) rats (Animal Experiment Center of Guangdong University of Chinese Medicine, License No: SCXK (Guangdong) 2013-0034) weighing 235–255 g were maintained under standard laboratory conditions with free access to food and water. The room temperature was maintained at 20–25 °C. The animals were housed in the same animal care facility on a 12:12-hr light/dark cycle. All experiments were approved by the ethics committee of Sun Yat-sen University of Medical Sciences. All efforts were made to minimize the pain and stress of the rats.

***Experimental groups and study design***

**Figure 1:**
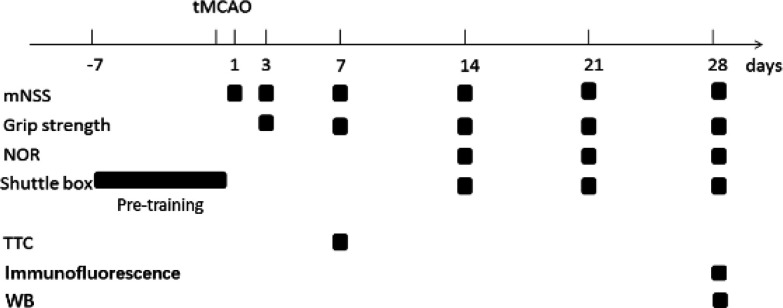
Study design of this research: The black boxes represent assessments or evaluations at different time points

One week before tMCAO, the rats underwent a trial related to the shuttle box test. Sixty rats with a 70% active avoidance response rate (AARR) underwent tMCAO. Subsequently, after undergoing Bederson’s assessment, the rats were randomly divided into the following four groups: tMCAO_60min_ group (n = 8), tMCAO_90min_ group (n = 11), tMCAO_120min_ group (n = 7), and sham group (n = 7). The rats were assessed according to the modified neurological severity score (mNSS) on days 1, 3, 7, 14, 21, and 28. Forelimb grip strength tests were performed on days 3, 7, 14, 21, and 28, while the shuttle box test was performed on days 14, 21, and 28. Another group of rats was randomly divided into four groups as follows: tMCAO_60min_ group (n = 8), tMCAO_90min_ group (n = 8), tMCAO_120min_ group (n = 7), and sham group (n=7). The novel object recognition test (NORT) was performed on days 14, 21, and 28. A final rat group was sacrificed to calculate the infarction volume through triphenyl tetrazolium chloride (TTC) staining on day 7 (n = 5). Figure 1 presents the study design.

***tMCAO procedures***

Stroke was induced using a transient filament occlusion tMCAO model in rats (20). There were three different occlusion times: 60 min, 90 min, and 120 min. To eliminate among-group systematic differences, we randomly assigned the animals to their respective experimental groups. The rats were anesthetized using 10% chloral hydrate (3 ml/kg, IP). We exposed the left common carotid artery (CCA) and bifurcation area using a midline anterior cervical incision and carefully separated it from the surrounding vagus nerve fibers. The external carotid artery (ECA) was sutured beyond the branches of the superior thyroidal and occipital arteries. A loose suture was placed around the internal carotid artery (ICA); furthermore, CCA and ICA were occluded using temporary atraumatic clips. The ECA was dissected. The filament (fishing line, Ø 0.25 mm) tip was blunted using a file and covered with silicon rubber (Ø 0.375 mm) to prevent vascular rupture. A filament was inserted into the ECA lumen. The ECA and filament were pulled backward until the ECA and CCA were parallel; subsequently, the filament was advanced into the ICA. Next, the suture around the ICA was tightened, followed by removal of the clip on the ICA. Finally, the filament was gently advanced until a slight resistance was felt 19–20 mm from the bifurcation to occlude the MCA origin. The resistance felt at approximately 10 mm was indicative of incorrect filament insertion, and the filament was pulled back and advanced again into the ICA. After 60, 90, or 120 min, the rats were re-anesthetized, and the suture was withdrawn to allow brain tissue reperfusion. The sham-operated animals underwent a similar surgical procedure except the filament was not advanced into the MCA.

***Behavioral tests***

*Bederson`s assessment*

After waking from anesthesia, the rats underwent neurological evaluation as described previously (21). Neurological function was scored on a 0–5 scale as follows: 0, no neurological deficit; 1, failure to fully extend the right forepaw; 2, circling to the right; 3, falling to the right; 4, no spontaneous walking and depressed consciousness level; and 5, death. In the tMCAO model, the exclusion criteria were score > 1-point, subarachnoid hemorrhage, or death.

*Modified neurological severity score (mNSS)*

mNSS is a composite of motor, sensory, balance, and reflex tests graded on a 0-18 scale, with a normal score of 0 and a maximal deficit score of 18 (22, 23). Regarding the injury severity scores, 1 point represents an inability to perform the test or a lack of the tested reflex, with higher scores reflecting more severe injuries. mNSS was performed on days 1, 3, 7, 14, 21, and 28 after MCAO induction by an investigator who was blinded to the treatments.

*Grip strength test*

Forelimb grip strength was measured using a grip strength test instrument (YLS-13 grip strength test instrument, JiaShi Scientific Instruments Company, Shanghai, China) as previously described (23). Briefly, the rat was held by its tail and pulled back gently until the front paw lost its grip. The maximum grip strength was recorded automatically. After grip strength evaluation of the affected forelimb, the unaffected forepaw was wrapped with tape. The grip strength was measured five times at each time point, and the mean value was obtained. An independent investigator who was blinded to the treatment conducted the grip strength test.

*Novel object recognition task (NORT)*

NORT assesses recognition memory based on the natural tendency of animals to preferentially explore novel, rather than familiar, objects. The experimental apparatus (XR-XX117 NORT system, Shanghai XinRuan) was a Plexiglas box (72 cm × 72 cm × 35 cm) with a black plastic floor. The objects for discrimination were square and triangular iron blocks. Animal behavior was recorded using a camera positioned directly above the box. The object recognition task was completed in three phases. During the habituation phase, the rats were allowed to freely explore the box without objects for 30 min. During the training phase, each rat was placed in the box with two identical objects (A and B) on the same spot and facing the same direction and was allowed to explore for 10 min. During the test phase, which was performed after 30 min, each rat was returned to the box for 10 min. The box contained the familiar object (B) from the habituation phase, whose position between both trials was consistent, and a novel object (C). To eliminate olfactory cues from previous mice, the box and objects were thoroughly cleaned using 70% ethanol. We recorded the time spent exploring individual and multiple objects. Object exploration was defined as pointing the nose toward the object at a distance ≤ 2 cm. Climbing or sitting on an object was not considered exploration. The object exploration time from the training and testing phases was presented as the discrimination index (exploration time of the novel object divided by total exploration time in the test phase), which indicated working memory(24).

*Two-way shuttle box test*

**Figure 2:**
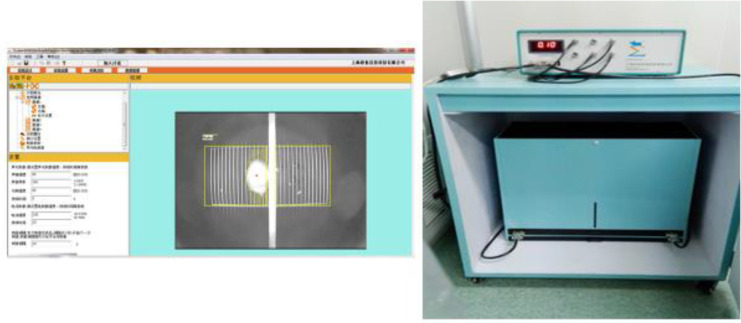
Two-way shuttle box operation interface window and the device of two-way shuttle box

The two-way shuttle avoidance task was performed using an XR-XC105 shuttle test video analysis system (Shuttle box system, Shanghai XinRuan, Figure 2). The box contains two identical compartments (226×213×350 mm) with a small hole between them for the rat to pass through. The floor of the box is a stainless-steel grid with electricity used as the unconditioned stimulus. There is a noise generator at the top (ringtone) and a light source on each side as conditioned stimuli. After being allowed 5 min for adaptation to eliminate investigatory behavior, the rats received a conditioned stimulus for 5 sec. If the rats fled to the safety zone (the other compartment) within 5 sec of the conditioned stimulus, the active avoidance reaction (AAR) was tested. Otherwise, 0.5 mA electrical stimulation was given for10 sec. Here, if the rat fled to the safety zone, the response was considered a passive avoidance reaction (PAR). Otherwise, we considered that there was no response. There was a 10-sec between-round interval. Each trial comprised twenty rounds. The general avoidance response (GAR) was the sum of PAR and AAR. AARR, which was calculated as the AAR/GAR ratio, was used to evaluate memory and avoidance learning (25, 26).

***Infarct volume measurement***

The infarct size was quantified at 7 days post-MCAO. The rats were decapitated; subsequently, the brain was removed and washed in ice-cold phosphate-buffered saline. The brain was maintained at -20 °C for 15 min and cut into 2-mm coronal sections using a brain slicer. The sections were incubated in 2% TTC for 20 min at 37 °C in the dark as described previously (27). TTC staining differentiates between live (red) and dead/dying tissue (white). The TTC-stained sections were transferred to 4% formaldehyde for fixation overnight at 4 °C. The infarct size was calculated using Image-Pro Plus 6.0 (Media Cybernetics, CA, United States). The infarct size (%) was defined as the difference in the undamaged tissue volume between the damaged and undamaged hemispheres.

***Immunofluorescence of the NgR1/RhoA pathway***

Brain sections were stained with the following primary antibodies at 4 °C overnight: rabbit anti-Nogo receptor (NgR; 1:100, Millipore), rabbit anti-RhoA (1:100, CST), rabbit anti-ROCK (1:100, Abcam), and rabbit anti-LOTUS (1:100, PL Laboratories). Subsequently, the sections were incubated with goat anti-rabbit (1:500, Alexa Fluor 488 IgG, CST) and goat anti-mouse (1:500, Alexa Fluor 488 IgG, CST) secondary antibodies.

***Western blotting analysis of the NgR1/RhoA/ROCK pathway***

A 2-mm strip adjacent to the peripheral infarct edge was collected in the hippocampus. We determined the protein concentrations using a bicinchoninic acid protein concentration determination reagent kit (Beyotime Institute of Biotechnology). The following primary antibodies were used: rabbit anti-Nogo receptor (NgR; 1:1000, Millipore), rabbit anti-RhoA (1:1000, CST), rabbit anti-ROCK (1:1000, Abcam), and rabbit anti-LOTUS (1:1000, PL Laboratories). Specific proteins were visualized using an enhanced chemiluminescence reagent kit (Millipore).

***Statistical analysis***

We expressed the results of mNSS and grip strength tests as the mean ± standard deviation (SD). NORT and shuttle box results are expressed as the mean± standard error (SE). Among-group differences were compared using one-way analysis of variance (ANOVA) followed by *post hoc* multiple comparisons using the least significant difference test. Behavioral evaluations were analyzed using repeated-measures ANOVA. In case sphericity assumptions were violated (Mauchly’s test; *P*<0.05), we applied the Greenhouse-Geisser correction. Between-group differences in immunofluorescence and western blot results were evaluated using an independent t-test, and the data are expressed as the mean (SEM). The correlation between ischemia occlusion duration and grip strength was analyzed using the Spearman test. Cognitive decline was defined as a score lower than the mean -2 SD of the sham group. All statistical analyses were performed using SPSS 20.0 statistical software. Statistical significance was defined as *P*<0.05.

## Results

***The infarct volume differed across the three ischemic groups***

**Figure 3:**
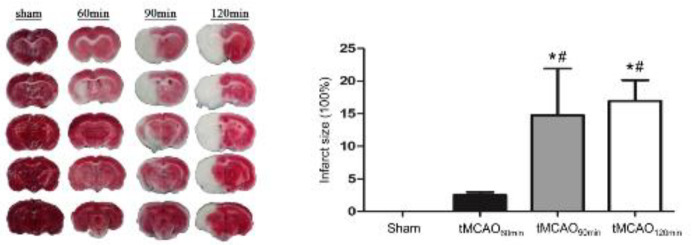
Comparison of infarct volume among the four groups of experimental rats*P<0.05 vs the sham group, #*P*<0.05 vs the tMCAO_60min_ group

Brain damage was localized to the cortex and striatum. The infarct sizes in the tMCAO_60min_, tMCAO_90min_, and tMCAO_120min_ groups were 4.64%, 18.13%, and 23.75%, respectively (Figure 3). There were significant differences in the infarct sizes among the ischemic groups (*P*<0.05).

***mNSS decreased over time in all ischemic groups***

The rats in the sham group did not show any neurological deficits. All three ischemic groups showed a significant increase in mNSS scores compared with those of the sham group during the 4-week observation period (*P*<0.05). As shown in **Table 1**, all ischemic groups showed a reduction in mNSS scores over time. One-way ANOVA showed significant among-group differences on day 1 (F = 5.623, df=3, *P*=0.009), day 3 (F = 11.154, df=3, *P*<0.001), day 7 (F = 7.971, df=3, *P*<0.05), day 14 (F = 6.869, df=3, *P*<0.05), day 21 (F = 8.118, df=3, *P*<0.05), and day 28 (F = 6.927, df=3, *P*<0.05). *Post hoc* comparisons revealed that compared with the tMCAO_60min _group, the tMCAO_90min_ group presented no significant difference at 7 and 14 days. Repeated-measures ANOVA revealed significant effects of time (F = 187.875, df=5, *P*<0.001) and treatment (F = 9.100, df=3, *P*=0.001) and no treatment × time interaction effect (F = 1.141, df=15, *P*>0.05)

***The recovery of grip strength in the affected forelimbs was time-dependent***

The grip strengths of the affected forelimbs are illustrated in Table 2. All ischemic groups had significantly decreased grip strength compared with that of the sham group at 3 d (*P*<0.05). There was no difference between the sham and tMCAO_60min_ groups at 7 d or between the sham and tMCAO_90min_ groups at 28 d. The tMCAO_120min_ group had decreased grip strength compared with that of the sham group during the 4-week observation period. Repeated-measures ANOVA revealed that there were significant effects of time (F=120.780, df=4, *P*<0.001) and treatment (F=11.631, df=3, *P*<0.001) and no treatment × time interaction effect (F=1.508, df=12, *P*>0.05).There was a negative correlation between ischemia occlusion duration and grip strength on days 3, 7, 14, 21, and 28 (r=﹣0.724, r=﹣0.715, r=﹣0.626, r=﹣0.681, r=﹣0.492, respectively; *P*<0.05).

***Ischemic rodents showed different performance in the novel object recognition task (NORT)***

One-way ANOVA of the discrimination index revealed no among-group differences in the three training phases (F = 0.269, df=3, *P*>0.05; F = 0.786, df=3, *P*>0.05; F = 1.844, df=3, *P*>0.05). This indicated among-group equivalence in object exploration at the three time points. During the testing phase (Figure 5), one-way ANOVA of the discrimination index revealed no among-group differences at 14 d and 21 d (F = 0.948, df=3, *P*>0.05; df=3, F = 1.468, df=3, *P*>0.05). At 28 d, one-way ANOVA revealed a significant group effect (F = 3.458, df=3, *P*=0.031). *Post hoc* comparisons revealed that compared with the sham group, tMCAO_90min_ and tMCAO_120min_ groups had a significantly reduced discrimination index (*P*=0.028 and *P*=0.006, respectively). There was no significant difference in the discrimination index between the sham and tMCAO_60min_ groups (*P*>0.05). Repeated-measures ANOVA revealed no significant time, treatment, or interaction effect (*P*>0.05). On day 28, cognitive impairment occurred in 25% of rats in the tMCAO_60min_ group, 50% of rats in the tMCAO_90min_ group, and 50% of rats in the tMCAO_120min_ group.

***Ischemic rodents had different performances in the two-way shuttle box task***

Repeated-measures ANOVA revealed a significant effect of treatment (F = 3.516, df=3, *P*<0.05) but not time (F = 1.057, df=2, *P*>0.05); moreover, there was no treatment × time interaction effect (F = 1.008, df=6, *P*>0.05). One-way ANOVA of AARR indicated a significant among-group difference at 14 and 21 d (F = 3.387, df=3, *P*<0.05; F = 4.221, df=3, *P*=0.05). However, there was no significant among-group difference at 28 d (F = 0.969, df=3, *P*=0.052). Compared with the sham group, the tMCAO_90min_ group presented a reduced AARR at 14, 21, and 28 d in *post hoc* comparisons (*P*<0.05), while the tMCAO_120min_ group showed a decreased AARR at 14 and 21 d. There was no significant difference in AARR between the tMCAO_60min_ group and sham group at 14, 21, and 28 d (Figure 6). On day 28, cognitive decline occurred in 25% of the tMCAO_60min_ group, 54% of the tMCAO_90min_ group, and 57% of the tMCAO_120min_ group.

***NgR1/RhoA/ROCK and LOTUS expression was increased in the ipsilateral thalamus***

Figure 7 shows the immunofluorescence results for the ipsilateral thalamus. NgR1, RhoA, and ROCK expression in the sham group was lower than that in the tMCAO_90min_ group (*P*<0.05). Compared with the control group, there was an increase in LOTUS expression in the ipsilateral thalamus (*P*<0.05) on day 28 post-tMCAO.

***NgR1/RhoA/ROCK and LOTUS protein levels increased in the ipsilateral thalamus***

NgR1, RhoA, and ROCK expression in the sham group was lower than that in the tMCAO_90min_ group (*P*<0.05). Compared with the sham group, the tMCAO_90min_ group showed increased LOTUS expression in the ipsilateral thalamus (*P*<0.05) on day 28 post-tMCAO. Figure 8 shows the western blotting results for the ipsilateral thalamus.

tMCAO: transient middle cerebral artery occlusion; mNSS: modified neurological severity score; NOR: novel objection recognition; TTC: triphenyl tetrazolium chlorid

**Table 1:** Comparison of mNSS among the three ischemic groups of experimental rats

Time (d)	tMCAO^60min^	tMCAO^90min^	tMCAO^120min^
1	6.50±2.01	8.81±2.04^*^	9.38±1.84^*^
3	4.20±1.23	5.82±1.54^*^	7.25±1.28^*^^#^
7	3.70±1.34	4.73±1.35	6.38±2.46^*^^#^
14	2.90±1.37	4.00±1.48	5.50±1.60^*^^#^
21	2.00±1.25	3.36±1.63^*^	4.75±2.06^*^^#^
28	1.30±0.67	2.73±1.68^*^	3.88±1.90^*^

* *P*<0.05 vs tMCAO_60min_ group, # *P*<0.05 vs tMCAO_90min _group

**Table 2:** Comparison of grip strength of the affected forelimb among the different groups (g) of experimental rats

time（d）	Sham	tMCAO_60min_	tMCAO_90min_	tMCAO_120min_
3	636.55±51.32	494.31±83.93*	431.13±116.61*	331.24±90.56*^#$^
7	678.83±74.32	601.65±62.67	502.80±141.07*	366.69±116.88*^#$^
14	794.31±53.19	705.94±126.06	653.45±126.24*	467.88±191.50*^#$^
21	842.35±55.02	777.75±70.24	712.40±83.65*	593.07±167.00*^#$^
28	873.80±83.95	833.68±93.76	819.17±74.53	684.20±114.15*^#$^

*P<0.05 vs sham, #*P*<0.05 vs tMCAO60min, $*P*<0.05 vs tMCAO_90min_

**Figure 4:**
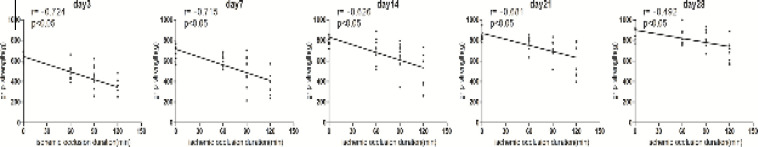
Analysis of the relationship between ischemia occlusion duration and grip strength on days 3, 7,14, 21, and 28 post tMCAO. There were negative correlation between ischemic occlusion duration and grip strength on this four time points

**Figure 5:**
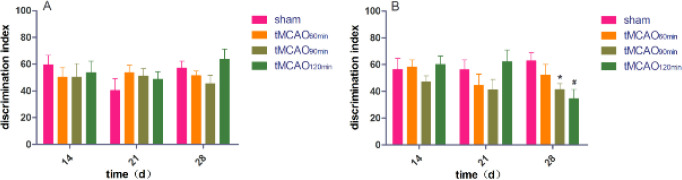
Comparison of the discrimination index from the novel object recognition test of experimental rats. A, training phase; B, testing phase. *P<0.05 vs the sham group, #*P*<0.05 vs the sham group

**Figure 6:**
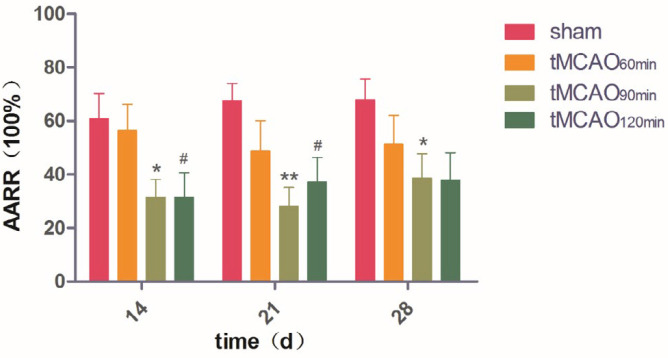
Active avoidance response rates (AARRs) of the different groups of experimental rats. *P<0.05 vs sham group; **P<0.01 vs sham group; #*P*<0.05 vs sham group

**Figure 7:**
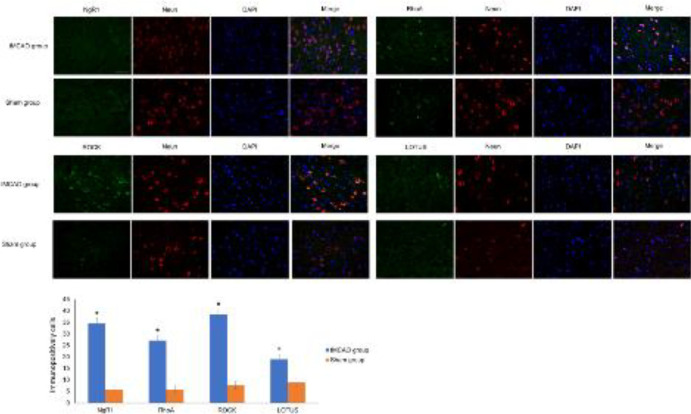
Immunofluorescence results for NgR1, RhoA, ROCK, LOTUS, and LGI1 expression in the hippocampus on the infarct side (A-E). The statistical immunofluorescence results (F): *P<0.05 compared with the control group

**Figure 8.**
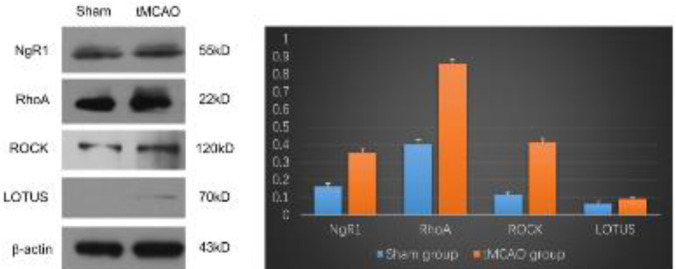
The Western blotting results for NgR1, RhoA, ROCK, LOTUS, and LGI1 expression in the hippocampus on the infarct side (A). The statistical WB results (B-F): *P<0.05 compared with the sham group of experimental rats. Nogo receptor-1; ROCK: RhoA-activated Rho kinase

## Discussion

This study aimed to further explore whether the NgR1 pathway is involved in cognitive dysfunction in tMCAO rats and to determine the optimal model for cognitive impairment assessment. Cognitive dysfunction has been reported in 62.6% of patients with stroke at three months after cerebral infarction (28). In the first poststroke year, 40% of patients present with cognitive impairment but do not meet the criteria for dementia (29). Furthermore, a progressive decline in cognitive function has been reported in patients with a large area of cerebral infarction (30). MCAO in rats is among the most widely used animal models of cerebral ischemia (31). However, there is no model for vascular dementia caused by cerebral infarction; moreover, the optimal MCAO protocol for assessing cognitive function remains unclear (32). We induced transient focal ischemia in rats by MCAO for 60 min, 90 min, and 120 min. By investigating the dynamics of sensorimotor and cognitive function, especially executive function, this study provides novel evidence regarding the optimal time for tMCAO when assessing cognitive function in SD rats.

The present study found that the ischemia duration was positively correlated with the NSS score and worsened grip strength of the affected forelimb in tMCAO rats (Table 1, Table 2, Figure 4). This suggests that the degree of motor and sensory impairment was affected by the ischemia-reperfusion duration (33). In contrast, another study (34) reported that functional outcomes were independent of artery occlusion duration. This could be because the 15-min ischemia duration before reperfusion was too short to cause visible functional differences. Regarding long-term outcomes, rats present with motor dysfunction even at one year post-tMCAO (35), as indicated by mNSS scores. Compared with the sham rats, the tMCAO_120min _rats had reduced grip strength. However, there were no significant differences in grip strength between the sham group and the tMCAO_60min_ and tMCAO_90min_ groups. This suggests that the motor function of tMCAO_60min_ and tMCAO_90min _rats is restored to nearly normal levels over time.

The 90-min and 120-min tMCAO groups had a larger infarct volume than the 60-min tMCAO group (Figure 3). All ischemic groups showed impaired sensorimotor function, as indicated by decreased grip strength (Table 2) and increased mNSS (**Table 1**). To minimize the interactive effect of different cognitive evaluations, we performed two cognitive assessments in the rat groups. The discrimination index in the NORT was indicative of working memory (36), which is an indicator of executive function. In addition, AARR was used to evaluate memory and avoidance. We found that only tMCAO_90min_ and tMCAO_120min_ groups showed cognitive impairment, as indicated by AARR (Figure 6) and discrimination index (Figure 5). These findings suggest that ≥ 90 min after tMCAO is the optimal time for cognitive function assessment in MCAO rats.

It remains unclear whether the rat MCAO model generates cognitive impairment. We found that tMCAO_90min_ and tMCAO_120min _rats showed cognitive impairment, especially impairment of executive function. However, researchers (10) reported that rats with tMCAO do not present learning and memory impairment. This finding could be attributed to the use of an elevated plus-maze for cognitive function assessment, which is more suitable for assessing anxiety-related rodent behavior. Researchers (9) reported minimal spatial memory deficits in this MCAO model and suggested that previously reported impairments in the water maze test were a result of sensory and motor deficits rather than memory deficits. Consistent with Xu *et al.* (37), we propose that the water maze test requires a high motor functioning level, which could result in false-positive results in cognitive studies. In contrast, the motor demands of the shuttle box test and NORT are relatively low. A study (38) reported that tMCAO rats showed impaired spatial learning and memory, with further progressive decline.

Regarding reperfusion time, a study (39) reported that 90-min MCAO in SD rats impaired spatial working memory, as assessed by a Y-maze test at 30 days post-MCAO. Using the same rodent model, Xu *et al*. (37) reported impaired passive avoidance performance in ischemic rats, which may be associated with endoplasmic reticulum stress-mediated neuronal apoptosis in the ipsilateral hippocampus within the first four days post-tMCAO. SD rats with 2-hour MCAO showed spatial memory and recognition memory dysfunction verified by the discrimination index of the NORT and Y-maze test during a 28-day follow-up period (40).In contrast, Andrews *et al*. (41) reported that SD rats subjected to 60-min MCAO performed worse than sham rats in the behavioral flexibility operant task during a 7-week observation period. This could be because the behavioral flexibility operant task is more sensitive for detecting cognitive deficits in MCAO rats. However, it was found to be challenging for a few rats that never learned the task (41).

Cognitive impairment caused by tMCAO could involve tMCAO-induced cortical injuries, including lesions in the neocortex and, more specifically, in the frontal cortex. Theoretically, MCAO does not damage the hippocampus, which is closely associated with memory and is mainly supplied by the posterior cerebral artery (42). However, MCAO induces ischemic damage in the MCA territory as well as deep areas beyond it, including the hippocampus, thalamus, and hypothalamus (43). Researchers (44) suggested that ischemic hippocampal, thalamic, and hypothalamic damage in the MCAO model is partially associated with small and deep artery occlusion, including in the anterior choroidal artery, lateral hypothalamic artery, and/or ventral thalamic artery. A study (45) reported that cognitive deficits caused by MCA territory stroke are not simply due to direct hippocampal damage; rather, they involve indirect alterations of hippocampus-thalamic connections. Another study (46) reported that bilateral loss of NMDA receptors, which probably reflects receptor down-regulation and internalization, could be attributed to stroke effects on cognitive function that are not solely attributable to infarction.

In the tMCAO_90min_ rat model, there was increased expression of NgR1 and downstream RhoA and ROCK (Figure 7). Furthermore, there was increased expression of the endogenous antagonist LOTUS in the ipsilateral thalamus (Figure 8), which could be involved in cognitive impairment mechanisms. Takase *et al*. (18) reported that transgenic LOTUS overexpression accelerates post-stroke neuronal plasticity compared with that in wild-type mice. Increased LOTUS expression after cerebral ischemia is a natural compensatory response to functional impairment; however, it is insufficient. RhoA induces axonal and dendritic retraction, as well as spine/synapse loss. Rho kinase (ROCK) is the key downstream effector. There is increasing evidence (47, 48) that suppressing the RhoA-ROCK signaling pathway could be a promising therapeutic target for improving information processing and memory storage after cerebral injury. There is a need for future studies to further explore this possibility.

## Conclusion

Taken together, our findings suggest that 90-min or 120-min MCAO in SD rats causes cognitive impairment, especially executive dysfunction impairment, as demonstrated by the shuttle box test and NORT. To minimize interference caused by motor dysfunction, we recommend a 90-min ischemia duration for studies employing cognitive-related behavioral testing. Moreover, we recommend scheduling the tests to be performed at least 28 days after surgery when the motor function has significantly recovered. Increased NgR1/RhoA/ROCK expression and insufficient increase in expression of their antagonist LOTUS could be involved in poststroke cognitive impairment.
